# Molecular landscape of borderline ovarian tumours: A systematic review

**DOI:** 10.1515/med-2024-0976

**Published:** 2024-06-07

**Authors:** Pawel Sadlecki, Malgorzata Walentowicz-Sadlecka

**Affiliations:** Medical Department, University of Science and Technology, Bydgoszcz, Poland; Department of Obstetrics and Gynecology, Regional Polyclinical Hospital, Grudziadz, Poland

**Keywords:** borderline ovarian tumours, molecular features, mutations, genetic mutations, *BRAF*, *KRAS*, *NRAS*, *ARID1A*, *CADM1*, *PIK3CA*, *CHEK2*, *CLAUDIN-1*, *ERBB2*, loss of heterozygosity, *PTEN*, microsatellite instability, B-CATENIN, E-CADHERIN, *BRCA 1*, *BRCA 2*

## Abstract

Borderline ovarian tumours (BOTs) show intriguing characteristics distinguishing them from other ovarian tumours. The aim of the systematic review was to analyse the spectrum of molecular changes found in BOTs and discuss their significance in the context of the overall therapeutic approach. The systematic review included articles published between 2000 and 2023 in the databases: PubMed, EMBASE, and Cochrane. After a detailed analysis of the available publications, we qualified for the systematic review: 28 publications on proto-oncogenes: BRAF, KRAS, NRAS, ERBB2, and PIK3CA, 20 publications on tumour suppressor genes: BRCA1/2, ARID1A, CHEK2, PTEN, 4 on adhesion molecules: CADM1, 8 on proteins: B-catenin, claudin-1, and 5 on glycoproteins: E-Cadherin. In addition, in the further part of the systematic review, we included eight publications on microsatellite instability and three describing loss of heterozygosity in BOT. Molecular changes found in BOTs can vary on a case-by-case basis, identifying carcinogenic mutations through molecular analysis and developing targeted therapies represent significant advancements in the diagnosis and treatment of ovarian malignancies. Molecular studies have contributed significantly to our understanding of BOT pathogenesis, but substantial research is still required to elucidate the relationship between ovarian neoplasms and extraneous disease, identify accurate prognostic indicators, and develop targeted therapeutic approaches.

## 1 Introduction

Borderline ovarian tumours (BOTs) exhibit intriguing characteristics that distinguish them from other ovarian tumours. Despite their unusual cellular structure and potential to spread, BOTs exhibit less aggressive behaviour than low- or high-grade serous ovarian cancers (LGSC and HGSC, respectively). This discrepancy has been observed since BOTs were first described by Taylor in 1929 [1]. In 2003, BOTs were officially recognized as a distinct group, and their most recent classification was published in 2014 by the World Health Organization [2]. BOTs occur in 1.8–4.8 per 100,000 women annually and comprise 10–20% of all epithelial ovarian cancers, with BOTs exhibiting a tendency towards higher prevalence within this broader group of cancers [3,4]. This unique type of non-invasive neoplasms is characterised by atypical growth of epithelial cells, nuclear atypia, and a moderate-level of mitotic activity that places a particular type between benign tumours and invasive cancers [5]. Notably, BOTs do not exhibit destructive stromal invasion, which differentiates them from other types of ovarian neoplasms. Although BOTs do not display destructive stromal invasion, they can be associated with microinvasion, lymph node implantation, and non-invasive or invasive peritoneal implantation [5]. Generally, BOTs display molecular and genetic alterations similar to those found in LGSCs. In some cases, a gradual progression has been observed from cystadenomas and BOTs to LGSCs [6]. Like invasive carcinomas, BOTs can be categorised into six histological subtypes based on the type of epithelial cells present. The most prevalent subtypes of BOTs are serous (50%) and mucinous (45%) BOTs, with endometrioid, clear cell, seromucinous, and borderline Brenner tumours being diagnosed less often [5,7].

BOTs are managed like invasive ovarian cancers, with a comprehensive staging process that guides the choice of the most appropriate surgical intervention. When compared to laparotomy, laparoscopy has not shown a negative impact in terms of the recurrence rate, the survival rate, or feasibility of surgically managing BOTs. If surgery without a risk of tumour rupture is possible, then the laparoscopic approach could be considered a feasible, and safe option to recommend over laparotomy [8]. Management typically involves a series of procedures, such as peritoneal washing cytology, hysterectomy, bilateral salpingo-oophorectomy, omentectomy, and the complete removal of visible peritoneal lesions. The frozen section (FS) plays an important role in determining the appropriate course of surgical management; however, the surgeon should be aware of the well-known limitations of FS. Specifically, while the diagnostic accuracy rate of FS remains high for benign and malignant ovarian tumours, it is relatively low for BOTs. Frozen samples tend to the underdiagnosis of BOTs as benign tumours in 25–30% of cases, and their improper identification as carcinomas in 20–30% of cases. More caution in the use of FS in the diagnosis of BOTs is therefore needed, especially in cases of bulky tumours, for which an intraoperative histology may lead to the misdiagnosis of some essential features (e.g., microinvasion, papillary variant, intraepithelial carcinoma, stromal microinvasion) [8].

BOTs often affect women in their reproductive years, so preserving fertility is a critical factor during treatment planning. Traditionally, fertility-sparing surgery has primarily been offered to patients with BOTs localised within the ovary. However, recent evidence suggests that some appropriately selected patients with advanced disease may also be eligible for fertility-sparing procedures without compromising their safety [8]. If a patient with BOT is eligible for a fertility-sparing treatment, a choice between cystectomy and unilateral salpingo-oophorectomy needs to be made in the case of unilateral tumours or between bilateral cystectomy and unilateral salpingo-oophorectomy with contralateral cystectomy in the case of bilateral tumours [9]. In patients with BOT, uterine-sparing surgery may also be considered; despite the increased risk of disease recurrence, the risk of death due to BOT does not increase in such cases [10]. While most patients with BOTs are usually diagnosed at early stages, the prognosis is still favourable even if the diagnosis is delayed [11]. An additional option for preserving fertility in BOTs consists of harvesting and cryopreserving oocytes prior to cytoreductive intervention [12].

Although the prognosis in patients with BOTs is typically promising, the risk of recurrence needs to be considered. The overall recurrence rate for BOTs is approximately 30%, but it may increase to 50% in patients diagnosed at advanced clinical stages [11]. Prognostic factors for recurrent and/or progressive disease include BOT type, patient age, stage, presence of invasive implants, microinvasion in the primary tumuor, and micropapillary architecture [11]. Notably, however, no single clinical or pathological feature, or a combination thereof, can be considered an accurate predictor of unfavourable outcomes. Nevertheless, several prognostic factors have been shown to increase the risk of recurrence, including the advanced stage of the disease at diagnosis, invasive peritoneal implants, and specific pathological features, such as intraepithelial carcinoma and a micropapillary growth pattern [13,14,15]. Unfortunately, treatment choices for patients with persisting or progressive disease are limited. Currently available chemotherapy schemes for invasive ovarian cancers have shown limited activity in treating BOTs [15], and given the lack of effective treatment options, managing persistent or progressive BOTs constitutes a challenge.

It should be noted that molecular changes found in BOTs can vary on a case-by-case basis, which warrants further research into the molecular landscape of these tumours. Identifying carcinogenic mutations through molecular analysis and developing targeted therapies represent significant advancements in the diagnosis and treatment of ovarian malignancies [6]. However, identifying patients with an increased risk of recurrence remains difficult. Continued research in this area is crucial for improving risk assessment and developing personalized treatment approaches. Against this background, the aim of the present systematic review was to analyse the spectrum of molecular changes found in BOTs and to discuss their significance in the context of the overall therapeutic approach.

## 2 Methods

The following systematic review was carried out in line with the established international standards and guidelines for systematic reviews (PRISMA). A detailed review protocol can be obtained from the author upon request. The systematic review included publications found in the following databases: PubMed, EMBASE, and Cochrane. Articles published between 2000 and 2023 were the subject of the research. Eligible studies were found using a combination of the following keywords: BOTs, molecular features, mutations, and genetic mutations. Searches were conducted on September 30, 2023. The language of the publications was limited to English and repeated items and articles without full text available were excluded from further analysis. Peer-reviewed observational studies and retrospective analyses were mainly included in the initial analysis.

**Figure 1:**
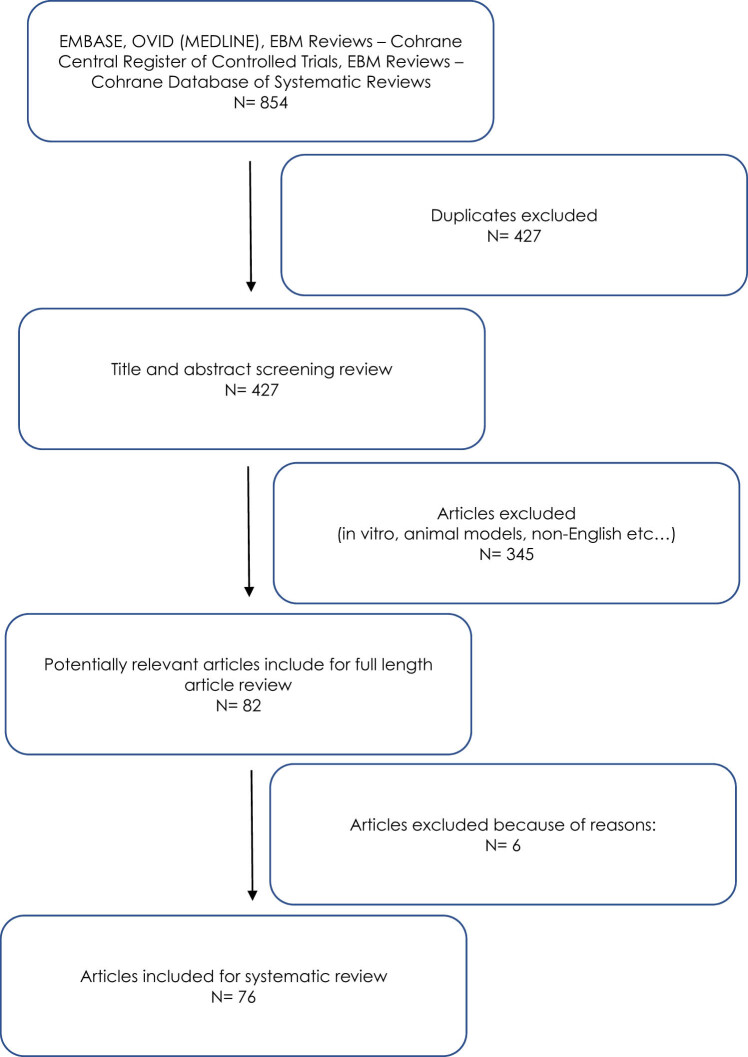
Flow diagram of study selection process.

After screening and obtaining the data, an analysis of the quality of the obtained articles was carried out. The Newcastle–Ottawa Scale was implemented to assess the quality of the included studies. A secondary search included examining the reference lists of all the included articles. After careful consideration, some specific publication types, such as editorials, comments, conference abstracts, case reports, abstracts, validation studies, and animal studies, were excluded from the analysis. The inclusion and exclusion criteria for this study are summarised in Table 1. A flow diagram illustrating the study selection process is presented in Figure 1.

**Table 1:** Inclusion and exclusion criteria for the study

**Inclusion criteria**
Key words: BOTs, molecular features, mutations, genetic mutations
Date: 2000–2023
Language: English
Participants: humans, adults
Type of the study: peer reviewed: original studies, observational studies, retrospective analyses
**Exclusion criteria**
Lack of availability of the full text of the article
Participants: animal studies
Type of the study: editorials, comments, conference papers, abstracts

## 3 Results

After the first stage of the search, a total of 854 studies were identified after excluding duplicates, of which 427 studies were included for further analysis. The analysis was limited to the following 15 molecular characteristics: *BRAF*, *KRAS*, *NRAS*, *ARID1A*, *CADM1*, *PIK3CA*, checkpoint kinase 2 (*CHEK2*), *CLAUDIN-1*, *ERBB2*, loss of heterozygosity (LOH), *PTEN*, microsatellite instability (MSI), B-catenin, E-cadherin, and *BRCA 1/2* mutation. Further analysis included solely English full-text articles presenting the results of studies in humans. Eventually, 76 out of 854 published studies were found to satisfy the inclusion criteria and were subject to the final analysis. After a detailed analysis of the available publications, the following selections qualified for the systematic review: 28 publications on the proto-oncogenes: BRAF, KRAS, NRAS, ERBB2, and PIK3CA; 20 publications on the tumour suppressor genes: BRCA1/2, ARID1A, CHEK2, and PTEN; 4 on the adhesion molecules: CADM1; 8 on proteins: B-catenin, claudin-1; and 5 on the glycoproteins: E-cadherin. In addition, in the final part of the systematic review, we included eight publications on MSI and three describing the LOH in BOT. Individual factors influencing the development, prognosis, and course of the disease are synthetically presented later in the article. In this study, the authors wanted to emphasize the fact that BOTs are not a homogeneous group of ovarian tumours in terms of histopathology and that differences between individual types of tumours also occur at the molecular level. A brief description of the mechanisms of action and clinical significance of the selected factors included in the analysis is presented in Table 2.

**Table 2:** Molecular changes in BOTs and their clinical significance

Factor	Function	Type of BOT	Mechanism	Clinical significance of mutation/presence
BRAF	Protooncogene	Serous	Inhibition of cell proliferation and differentiation	Lower risk of progression
KRAS	Oncogene	Serous	Higher cell proliferation and differentiation	Aggressive phenotype, higher risk of progression
NRAS	Oncogene	Serous	Cell polarity, proliferation, differentiation, adhesion, migration, apoptosis	Advancing tumours to more invasive forms
ARID1A	Suppressor gene	Endometrioid clear cell seromucinous	Chromatin remodeling	Loss of expression more aggressive behavior
CADM1	Adhesion molecule	Serous	Adhesion	Loss of expression more aggressive behavior
PIK3CA	Oncogene	Serous seromucinous endometrioid	Cell growth, metabolism, proliferation, survival, motility, and invasion	Aggressive phenotype, higher risk of progression
CHEK 2	Suppressor gene	Serous	Proliferation, cell cycle regulation, DNA repair	Earlier age of diagnosis, diminished survival rate
Claudin-1	Membrane protein	Serous	Overexpression	Activation of MAPK pathway
ERBB2	Protooncogene	Serous seromucinous	Cell proliferation	Activation of MAPK pathway
LOH	Loss of heterozygosity	Serous mucinous	Deletion of chromosomal regions	Marker of transformation higher risk of progression
PTEN	Suppressor gene	Endometrioid	Regulating transcription, translation, cell cycle progression, induction of apoptosis	Tumour progression
MSI	Microsatellite instability	Serous	Abnormal length of microsatellite repeats	Possible progression to invasive form
B-catenin	Multifunctional protein	Endometrioid	Adhesion, signal transduction	Poor prognosis early event of malignization
E-cadherin	Transmembrane glycoprotein	Serous	Promoting EMT-facilitating metastases	Aggressive phenotype, higher risk of progression
BRCA 1/2	Suppressor gene	Serous	DNA repair	Earlier diagnosis of BOT

## 4 Discussion

### 4.1 BRAF

The *BRAF* oncogene is a well-known proto-oncogene present in normal cells that is capable of transforming into an oncogene under various stimuli. The transformation leads to changes in the oncoprotein’s quantity or quality of the oncoprotein, which disrupts normal metabolic processes and promotes the shift towards cancerous cells [16]. Mutations in the *BRAF* gene are commonly observed across multiple cancers, with around 95% of the anomalies being T1799A point mutations, which are also known as BRAFV600E [16]. *BRAFV600E* is associated with abnormal activation of the protein encoded by the *BRAF* gene, which initiates downstream signal transduction pathways that enhance cell proliferation and alter differentiation [17,18]. The *BRAFV600E* mutation has been identified as a prevalent genetic alteration in serous borderline tumours (SBOTs) and LGSCs and is found in up to 40% and 5–10% of these tumours, respectively [19,20]. Interestingly, patients with *BRAFV600E*-mutated SBOTs were shown to have a lower risk of progression to invasive serous carcinoma, which implicates the possible protective role of this mutation in the transition to more aggressive ovarian cancers [20,21]. Additionally, SBOTs harbouring *BRAFV600E* mutations were demonstrated to have a distinct cellular morphology, thereby potentially representing cellular senescence, which refers to a state of growth arrest in response to stress [22]. Intriguingly, LGSCs, which are more advanced malignant tumours than SBOTs, often do not display this senescence-associated morphology, which points to a potential mechanism for their aggressive behaviour and progression. Overall, these findings highlight the significance of *BRAF* mutations, especially *BRAFV600E*, in the development and progression of SBOTs and LGSCs [23]. Understanding the molecular characteristics associated with *BRAF* mutations may provide a better insight into the biology of these ovarian cancer subtypes and help establish more effective prognoses and targeted therapeutic interventions.

The discovery of recurrent non-*V600 BRAF* driver mutations in various tumour types has led to a new classification system based on the results of preclinical studies [24]. In this system, *BRAF* mutations are divided into three classes: Class I, including V600E, V600D, V600K, and V600R mutations, with high kinase activity and RAS-independent monomer signalling; Class II mutations with intermediate kinase activity and RAS-independent dimer signalling; and Class III mutations, with either lack or exhibit impaired kinase activity and rely on RAS-dependent heterodimer formation for downstream signalling. This classification system reflects the tumour’s response to MAPK pathway inhibitors, which are commonly used to treat malignancies with *BRAF* mutations. While Class I mutations are more sensitive to MAPK pathway inhibitors, Class II and Class III mutations may show, a varying degrees of resistance or reduced sensitivity [25]. In conclusion, the knowledge of specific *BRAF* mutations and their clinical implications may facilitate the selection of personalised treatment strategies for patients with *BRAF*-mutated tumours. This is because accurate molecular classification and profiling are crucial for tailoring effective therapeutic approaches.

### 4.2 KRAS

Mutations in the *BRAF* and *KRAS* genes are the most common genetic abnormalities found in SBOTs and LGSCs [26]. Interestingly, LGSCs with *KRAS* mutations tend to exhibit higher aggressiveness and are more likely to recur than malignancies with *BRAF* mutations [26]. According to the literature, *KRAS* and *BRAF* mutations are detected in approximately one-third of BOTs and one-third of LGSCs. However, the co-occurrence of *KRAS* and *BRAF* mutations has never been observed within the same tumour, which implies that these malignancies harbour either *KRAS* or *BRAF* anomalies [27]. Both *KRAS* and *BRAF* play crucial roles in the RAS-RAF-MEK, ERK, and MAPK signalling pathways, which are pivotal for the regulation of cell proliferation [28]. The *RAS* oncogene family comprises three principal members: *KRAS*, *HRAS*, and *NRAS*, all of which have been linked to the development of various human malignancies [28]. *KRAS*, located on chromosome 12p12, encodes a 21-kD protein (p21RAS) essential to MAP-kinase signal transduction that controls cellular proliferation and differentiation [29]. Mutations in *KRAS* lead to constitutive activation of this signal transduction pathway, resulting in uncontrolled cell proliferation and differentiation [30]. The incidence of *KRAS* mutations in SBOTs is similar to that in LGSCs and ranges between 19 and 54.5% [31]. SBOTs without a *BRAF* mutation may progress to LGSCs because of *KRAS* mutations or other genetic alterations. Knowledge of these genetic aberrations is crucial for a better understanding of SBOT biology and perhaps also developing more effective targeted treatment strategies.

Another intriguing observation is the finding that patients with the *KRAS G12V* mutation had shorter overall survival rates than those without this mutation. This suggests that SBOTs with the *KRAS G12V* mutation might be a more aggressive phenotype of BOTs that may recur as LGSCs [29]. This notion is supported by the results of a study that included over 3,000 colorectal cancer samples, and in which the *KRAS G12V* mutation was the only one among 12 different mutations in *KRAS* codons 12 and 13 associated with poor overall survival.

While surgery remains a cornerstone of SBOT treatment, one direction of ongoing research is the identification of molecular alterations that could facilitate the choice of other therapeutic options. Unfortunately, published data on the influence that molecular characteristics have on the outcomes of SBOT treatment are limited. Nevertheless, sparsely available evidence suggests that SBOT cell lines with *KRAS G12V* mutations might be more responsive to AZD6244 (selumetinib) compared to cell lines with wild-type *KRAS* [32].

### 4.3 NRAS

*NRAS*, a well-known oncogene implicated in some malignancies, such as leukaemia and melanoma, is a part of the human *RAS* gene family, along with *HRAS* and *KRAS* [33]. This particular family, which includes the two *KRAS* variants of *KRAS4A* and *KRAS4B*, encodes closely related proteins consisting of approximately 188–189 amino acids [34]. These RAS proteins serve as GDP/GTP-regulated switches on the inner cell membrane and are thus crucial to the transmission of extracellular signals and the governance of vital intracellular signalling pathways. The latter pathways play pivotal roles in fundamental cellular processes, such as cell polarity, proliferation, differentiation, adhesion, migration, and apoptosis [35]. Mutations in the *NRAS* gene result in the constant activation of intracellular signalling through some pathways, such as RAS–RAF–MAPK and phosphatidylinositol 3-kinase (PI3K)/protein kinase B (AKT) [35]. Regarding cancers, some shared mutations, specifically *KRAS/BRAF* and *TP53/BRCA* mutations, have been found in low- and high-molecular grade tumours, respectively [36]. However, not all tumours harbour these mutations, which implies that other, not yet fully defined, pathway-related events, including *NRAS* mutations, could be involved. A study of serous ovarian tumours demonstrated that the presence of RAS pathway mutations might be associated with variable pathogenic effects. The early occurrence of co-mutations implies that *KRAS* and *NRAS* could play a role in the regulation of distinct cellular functions, thereby potentially producing a synergistic effect, as *KRAS* impacts proliferation, and *NRAS* influences cell survival [37]. Mutations in either *KRAS* or its homologue, *NRAS*, were found in 21 and 26% of LGSCs, respectively. Notably, *NRAS* mutations are present in SBOTs that show traits of transformation into ovarian cancer but are absent in those lacking the transformation features [38]. In contrast, *KRAS* and *BRAF* mutations can be found in early-stage ovarian malignancies, even before the SBOTs stage, and additional driving events, including *NRAS* mutations, are thought to expedite the disease’s progression [38]. These findings suggest that *NRAS* might act as a significant oncogenic driver in the progression of SBOTs into more invasive forms [38].

The occurrence of *NRAS* mutations in SBOTs with invasive characteristics highlights the potential role of this gene in ovarian cancer pathogenesis and warrants further research in this matter. The early occurrence of *KRAS* and *NRAS* co-mutations points to the distinct role of these genes in cellular function and ovarian cancer progression [37,38]. Nevertheless, further research is needed to understand the exact roles of these mutations and their potential synergistic effects in ovarian cancer development, especially in the context of potential personalised therapies targeting *NRAS* or its downstream effectors.

### 4.4 ARID1A

*ARID1A* is a tumour suppressor gene that is frequently altered in ovarian neoplasms linked to endometriosis, such as clear cell and endometrioid carcinoma [39,40]. Previous studies have documented the presence of somatic *ARID1A* mutations in 46–57% of ovarian clear cell carcinomas, 30% of ovarian endometrioid carcinomas, and 40% of uterine endometrioid carcinomas [40,41]. Molecular alterations found in seromucinous borderline tumours (SMBOTs) strikingly differ from those observed in other borderline (serous and mucinous) ovarian tumours. In a study involving 24 SMBOTs, the loss of *ARID1A* expression was found in approximately 33% of the cases (8 out of 24), including one case of synchronous endometriosis [42,43].

*ARID1A* encodes the BAF250a protein, which is critical to the formation of switch/sucrose nonfermentable chromatin remodelling complexes [39]. Most *ARID1A* mutations are nonsense, frameshift, or in-frame mutations that lead to loss of BAF250a expression. Thus, the absence of BAF250a immunoreactivity is indicative of *ARID1A*-inactivating mutations in preserved tissues [39,44]. According to Ayhan et al., 66% of ovarian endometrioid and clear cell carcinomas presented with *ARID1A* (BAF250a) expression loss [43]. Seromucinous tumours are not often associated with endometriosis, and their limited WT1 expression disconnects them from serous neoplasms [45,46]. Meanwhile, the available evidence points to the loss of *ARID1A* expression as a potential link between seromucinous tumours and endometrioid/clear cell neoplasms [45]. This feature differentiates seromucinous tumours from serous tumours, as the latter do not present with *ARID1A* expression loss, nor do they harbour any mutation in this gene. The results of morphological, immunohistochemical, and genetic analyses confirm that seromucinous tumours are not composed solely of serous and mucinous epithelium, thus debunking prior misconceptions [46].

To summarize, seromucinous tumours differ from serous tumours and appear to be linked more closely to endometrioid and clear cell neoplasms through the loss of *ARID1A* expression and other molecular traits.

### 4.5 CADM1

CADM1, an adhesion molecule from an immunoglobulin superfamily, is recognised as a tumour suppressor that plays a significant role in the progression and spread of various epithelial malignancies, especially in squamous cell carcinomas of the lungs, head, neck, oesophagus, and cervix [47,48]. CADM1’s extracellular domain engages with HER2 on the cell surface, thereby regulating downstream STAT3 activity. This interaction effectively restrains tumour growth and diminishes the potential for metastatic spread [48,49]. However, alterations within the CADM1/HER2/STAT3 axis in breast and lung adenocarcinomas have been shown to be linked to the more aggressive phenotypes of these malignancies [49,50]. According to the literature, CADM1 is expressed in all serous cystadenomas and in up to 83.33% of SBOTs. While benign serous cystadenomas and SBOTs exhibit the overexpression of CADM1, the expressions of HER2 and STAT3 in these tumours are reported to be either scant or low [51]. Interestingly, an opposite expression pattern, with reduced CADM1 expression and overexpression of HER2 and STAT3, was observed in malignantly transformed LGSCs and HGSCs [50,51]. Notably, the expression of CADM1 was shown to be weaker or absent in malignant LGSCs and HGSCs. The expression of CADM1 was also demonstrated to correlate inversely with HER2 and STAT3 expressions in serous ovarian tumours alongside evidence that associated the loss of CADM1 expression with aggressive tumour behaviour and lymph node metastases [51]. These findings highlight the potential role of CADM1 expression loss in the pathogenesis of serous ovarian tumours, which implies that this parameter could serve as a novel molecular marker for identifying the disease and monitoring its progression. Furthermore, a better understanding of the CADM1/HER2/STAT3 axis and its implications for tumour pathogenesis might inspire new perspectives on how to develop more effective, targeted therapeutic interventions.

### 4.6 PIK3CA

The PI3K–AKT signalling pathway has been shown to be activated in multiple cancers and is recognized as an essential regulator of cell growth, metabolism, proliferation, survival, mobility, and invasion [51,52]. More specifically the *PIK3CA* oncogene was identified as the most frequently mutated gene in uterine endometrioid and breast carcinomas. The presence of *PIK3CA* mutations has also been described in BOTs, whether serous, seromucinous, or endometrioid [53]. The role of *PIK3CA*, *BRAF*, and *ERBB2* mutations in the pathogenesis of LGSCs with synchronous SBOTs was the subject of a comprehensive study, where in one patient, *PIK3CA*, *BRAF*, and *ERBB2* mutations were found solely in LGSC but not in synchronous SBOT, whereas in another patient, *PIK3CA* mutations were detected in both LGSC and SBOT. The results of this study imply that *PIK3CA* and *ERBB2* mutations are significant events occurring during the transformation of serous cystadenoma to SBOT and further to LGSC [54]. Interestingly, the frequency of *PIK3CA* mutations in LGSCs/SBOTs in the Japanese population appears to be notably higher than that in Western patients [54]. This implies that the *PIK3CA* mutation might play a primary role in developing LGSCs in Japanese patients, with *BRAF* or *ERBB2* mutations acting as secondary factors [53,54]. Considering these findings, targeting the PIK3CA/AKT pathway through molecular therapies appears to be a potentially promising treatment for LGSC in Japanese patients. Understanding mutational patterns and their roles in LGSC progression might open up even more pathways to the development of more effective, personalised therapeutic approaches [54].

### 4.7 CHEK2

*CHEK2*, located at 22q12.1, is a critical tumour suppressor gene that encodes a serine-threonine kinase called the CHEK2 protein [55,56]. The latter, which acts as an anti-oncogene, interacts with other proteins, including P53, to control the cell cycle and to avert uncontrolled cellular proliferation. CHEK2 plays a crucial role in DNA repair and triggers cell cycle halt or apoptosis in response to DNA damage. The presence of mutations within the CHEK2 gene has been shown to be associated with a plethora of malignancies, both hereditary and non-hereditary [56,57], including breast, prostate, lung, colon, kidney, and thyroid cancers. The available evidence also points to the potential involvement of CHEK2 mutations in BOTs. One study demonstrated that patients with BOTs carried a common missense mutation (c.470T>C) within the CHEK2 gene [56,57]. The role of that mutation was further analysed in patients with ovarian cystadenomas, BOTs, and ovarian cancers. Ultimately, the findings revealed a substantial link between the presence of the mutation and the risk of non-invasive tumours, along with a borderline significant correlation with LGSC risk. Interestingly, the link with the CHEK2 missense mutation (c.470T>C) was confirmed to be statistically significant in the case of BOTs but not in ovarian cancer. The fact that the study included a higher number of low-grade ovarian cancer cases than tested previously might point to a discrepancy in the results of previous research and implies that the CHEK2 missense mutation (c.470T>C) might not actually contribute to ovarian cancer risk. This idea stands alongside the results of other studies in which CHEK2 mutations were shown to be associated with increased risks of prostate and breast malignancies but not ovarian cancer risk [58]. Furthermore, the available evidence suggests that the CHEK2 missense mutation might be associated with a two-fold increase in BOT risk. Additionally, a link has been found between the presence of the CHEK2 missense mutation (c.470T>C) and an earlier age at the diagnosis of BOT [57,58]. While overall survival rates in BOT patients are generally more favourable than in those with ovarian cancer, the 10-year survival rates in the mutation carriers were shown to be approximately 10% lower than those in non-carriers.

In summary, the available evidence suggests that while the CHEK2 missense mutation (c.470T>C) might be involved in BOT pathogenesis, it does not significantly influence the development of low-grade ovarian cancer. Generally, the mutation appears to be associated with an earlier age at the BOT diagnosis and a somewhat diminished 10-year survival rate [In summary, available evidence suggests that the CHEK2 missense mutation (c.470T>C) might be involved in BOT pathogenesis but does not significantly influence the development of low-grade ovarian cancer. The mutation appears to be associated with an earlier age at the BOT diagnosis and a somewhat diminished 10-year survival rate [57,58]. However, more research is needed to fully understand the implications of CHEK2 mutations for ovarian cancer and BOTs.

### 4.8 Claudin-1

Claudins are a family of integral membrane proteins situated within tight junctions, that are crucial for signal transmission and cellular transport [59]. The compromised integrity of tight junctions has been shown to play a role in the pathogenesis of solid tumours. Notably, the expression of claudin protein in tumour cells was demonstrated to differ from that in adjacent normal cells; while some malignancies showed a decrease in claudin protein expression, its expression in other tumour types was increased or mis-localised. Over 20 various claudins have been identified and characterised thus far. The role of these proteins in cancer pathogenesis is intricate, as they can either facilitate or inhibit tumour growth [60,61,62]. Furthermore, claudin expression has been shown to have prognostic value in various malignancies, which points to its function as a potential therapeutic targets. In particular, claudin-1 has been identified as a prognostic factor in multiple cancer types. For instance, one study analysed the link between claudin-1 and clinicopathological parameters in BOTs and revealed a significant association between the robust expression of this particular protein and certain histological characteristics [61,62]. Specifically, the overexpression of claudin-1 was demonstrated to correlate with the presence of peritoneal implants and a micropapillary pattern, both of which have been recognised as unfavourable prognostic factors in BOT. Moreover, claudin-1 overexpression in BOT appears to be associated with the activation of the mitogen-activated protein kinase pathway [61,62]. The latter observation justifies further research on the prognostic value of claudin-1 in BOT and its role as a potential therapeutic target interfering with the mitogen-activated protein kinase pathway.

### 4.9 ERBB2

The family of epidermal growth factor receptor (EGFR) proteins includes ERBB1 (EGFR), ERBB2, ERBB3, and ERBB4. The ERBB family of receptors has been studied extensively, given their role in the normal development of ovarian follicles and their regulation of the growth of the ovarian surface epithelium [63,64]. For instance, ERBB2, a member of the EGFR family, activates the PI3K/AKT and MAPK/ERK pathways to regulate cell proliferation, migration, and differentiation [64], and overexpression of the ERBB2 proto-oncogene occurs in 11–30% of epithelial ovarian cancers (EOC) [65,66]. The expression of ERBB2 has traditionally been evaluated by immunohistochemistry, with inconsistent prognostic results for epithelial ovarian cancer [66]. While increased ERBB2 expression intensity was shown to be associated with decreased median and overall survival in EOC in some studies, other authors found no significant relationship between the expression of this protein and survival rates. *ERBB2* mutations have been found in SBOTs, SMBOTs, and mucinous BOTs (MBOTs) [66,67], and *ERBB2* was even identified as one of the most prevalent mutant oncogenes in SMBOTs in the Japanese population [65,66,67]. Generally, *ERBB2* mutations are found in approximately 6% of SBOTs and typically do not co-exist with *KRAS* or *BRAF* mutations. According to the most widely accepted hypothesis, alterations in any of these three genes, all serving as upstream regulators of the MAPK pathway, can activate the latter pathway, with the result being uncontrolled cell proliferation. Thus, the available evidence suggests that each BOT might have its own distinct molecular mechanism of carcinogenesis.

### 4.10 LOH

The origin of additional diseases associated with ovarian tumours, specifically in terms of whether they result from tumour spread (monoclonal origin) or develop independently (multifocal origin), is a matter of ongoing debate. Previous research on the LOH in ovarian cancer identified multiple regions with a high frequency of LOH, which pointed to the potential involvement of tumour suppressor genes [68,69]. LOH has been observed across various chromosome arms at varying frequencies. Given that some benign and borderline tumours may represent early stages of ovarian carcinogenesis, research on these precursor lesions could provide an insight into the required accumulation of genetic events needed for the transformation of normal ovarian epithelium into benign, borderline, malignant, or metastatic tumours [69]. The presence of LOH at D11S860 and D7S522 in borderline cystadenomas and stage II invasive tumours suggests that these somatic genetic events might occur relatively early in ovarian carcinogenesis. Additionally, the frequent occurrence of LOH in *BRCA1*-containing loci in invasive tumours raises a question about the potential role of benign and borderline lesions with LOH at these specific loci as precursors of the invasive disease; specifically, it has been implied that 17q and 18q LOH in benign tumours might serve as a risk factor for malignant transformation [70]. However, the results of research on X-chromosome inactivation and LOH studies are inconclusive, which has complicated the determination of clonality. Mutational analysis, which was expected to clarify whether extra-ovarian diseases have a monoclonal or multifocal origin, has not provided a definitive answer. Given the discrepancies between the results of X-chromosome inactivation research and LOH studies and the inherent limitations of these methods, alternative approaches are needed to address the issue of ovarian tumour clonality more definitively.

### 4.11 PTEN

*PTEN*, which functions as a tumour suppressor gene, has often been reported to be lost in endometrioid carcinoma [71]. Genomic alterations or mutations within the *PTEN* can emerge prior to the malignant transformation of endometroid foci, which potentially disrupts the inhibitory function of this gene and initiates antiapoptotic pathways [72]. Located on chromosome 10q23, *PTEN* encodes a lipid phosphatase that acts as a negative regulator of PI3K by dephosphorylating phosphatidylinositol 3,4,5-trisphosphate [PI(3,4,5)P3]. Phosphorylated PI(3,4,5)P3 triggers the activation of the proto-oncogene protein kinase B (PKB/AKT), which in turn inhibits the apoptotic pathways, and activates mechanistic targets of rapamycin or deactivating forkhead family proteins [72,73]. The PTEN-mediated dephosphorylation of PI(3,4,5)P3 leads to the activation of the apoptotic pathway, with the resultant restrain of the PI3K/AKT signalling.

*PTEN* mutations are frequently found in endometrial and ovarian cancers, especially the endometrioid subtype, and often in early-stage tumours. These mutations are commonly detected in endometrioid BOTs (EBOTs) or even in the areas of non-atypical endometriosis. *PTEN* mutations have been reported in approximately 12% of EBOTs [73,74,75]. Importantly, EBOTs are also the tumours in which PI3K/AKT pathway activation is observed particularly often, thereby implying that early-occurring *PTEN* mutations might be involved in the development of these lesions [74]. However, the formation of an invasive carcinoma may require additional genetic changes to activate the PI3K/AKT pathway. Indeed, ovarian endometrioid carcinomas, which are genetically stable and originate from EBOTs, exhibit mutations not only in *PTEN* but also in other genes, such as *ARID1A*, *PIK3CA*, and *TP53*. The available evidence indicates that although LOH at the *PTEN* locus is infrequent in endometriosis, somatic *PTEN* gene mutations can be quite common in solitary endometriotic cysts [74,75]. Indeed, reduced PTEN protein expression has actually been reported in some cases of endometriosis [75].

In conclusion, the available data on the role of *PTEN* as a tumour suppressor and its mutation dynamics within endometrioid carcinomas and EBOTs highlight the importance of this gene in ovarian carcinogenesis. Thus, a better understanding of the molecular events associated with the functional loss of *PTEN* might inspire the development of new targeted therapies for ovarian malignancies.

### 4.12 MSI

Microsatellites, a notion used to describe the short repeating sequences of DNA bases found across the genome, can vary in length from tens to hundreds of bases. These regions are particularly susceptible to mutations, such as the insertion or deletion of repeating units, during DNA replication [76,77]. The alteration in microsatellite length is referred to as Microsatellite Instability (MSI). MSI occurs due to defects in DNA mismatch repair mechanisms, which disrupt DNA replication, resulting in increased mutation rates and contributing to the development of tumours with replication errors [76,77,78]. Examples of tumours with the replication error phenotype are colon and endometrial carcinomas that exhibit minimal chromosomal abnormalities but still display MSI, thereby indicating defects in mismatch repair [78,79].

It is still unclear whether MSI could also be involved in the pathogenesis of BOTs. The results of research on MSI in BOTs vary considerably, with the MSI rates ranging from none to 30%; these discrepancies could be attributed to a plethora of factors, among which are the type and number of markers used for detecting MSI and the criteria for defining the latter [78,79,80,81]. BOTs typically display limited chromosomal abnormalities, but the extent to which MSI plays a role in the development of these lesions remains unclear. Some authors have detected MSI in up to 27–30% of BOTs, whereas others found no evidence of MSI [6,81,82]. These results seem to support the dualistic model concept in which BOTs and invasive ovarian cancers follow distinct pathways that possibly originate from different cell clones with unique genetic modifications [82]. Moreover, research has demonstrated significant differences in MSI found in serous BOTs and serous invasive EOC, especially on chromosome 3 [81,82]. Instead of a gradual increase in allelic imbalance, observed during the progression of non-invasive to invasive micropapillary serous carcinomas in BOTs, HGSCs present with higher levels of allelic imbalances, which are found even in smaller primary tumours [82]. These findings suggest that MSI and chromosomal instability may play pivotal roles in the formation of BOTs and their progression from the normal ovarian epithelium. While shared chromosomal aberrations are common in both BOTs and high-grade tumours, their exact functions in these two types of lesions remain unclear.

### 4.13 β-Catenin

β-Catenin, a multifunctional protein, plays a crucial role in two important biological processes: cell-cell adhesion and signal transduction involving transcriptional activation. The involvement of β-catenin in cell adhesion is a well-established phenomenon, particularly within the adherent junctions of epithelial cells, where the cytoplasmic domain of E-cadherin orchestrates a peripheral protein complex essential for adhesion [83]. The activation of the WNT signalling pathway, which includes *CTNNB1* mutations, has been associated with fibrotization of the surrounding area, enhances proliferation, and promotes implantation or invasion [83,84]. This phenomenon was observed in cases of endometriosis, with consequences being proportional to the extent of the protein defect. *CTNNB1* gene mutations were found in various malignancies, such as pulmonary, breast, colorectal, endometrial, and ovarian cancers, the latter including endometriosis-associated ovarian malignant tumours. Immunohistochemistry revealed the expression of β-catenin in 61.2% of epithelial ovarian cancer patients, both endometriosis-free and those with concomitant endometriosis [74,84]. Mutations of the β-catenin gene and β-catenin overexpression are commonly found in ovarian endometrioid carcinomas, with approximately 50% of these malignancies exhibiting β-catenin alterations [74,84]. Moreover, up to 90% of EBOTs, among them endometrioid borderline carcinomas, were shown to harbour the β-catenin gene mutations [74]. Those findings suggest that *CTNNB1* mutations may represent an early event in the malignant transformation of certain ovarian tumours. Specifically, mutations of the β-catenin gene appear to be common in the early stages of endometrioid ovarian carcinoma development [85].

The fact that most endometriosis-associated tumours are low-grade lesions with relatively favourable prognoses warrants further research into the role of β-catenin as an early marker for endometroid ovarian carcinoma. Generally, the pattern of β-catenin expression varies across different histological types of ovarian carcinomas. The activation of the APC-B-catenin-Tcf signalling pathway, which is associated with the presence of an oncogenic β-catenin mutation, is a characteristic feature for a subset of endometrioid carcinomas with nuclear β-catenin expression and a favourable prognosis, many of which originate from benign tumours or BOTs. In turn, endometrioid carcinomas that display the membrane expression of β-catenin solely appear to be a distinct subgroup of malignancies not associated with the β-catenin signalling pathway that likely harbour a poorer prognosis.

### 4.14 E-cadherin

E-cadherin, a calcium-dependent transmembrane glycoprotein (120 kDa), serves as a tumour suppressor that prevents the progression and spread of various epithelium-derived carcinomas [74]. E-cadherin is encoded by the cadherin-1 (CDH1) gene, which is located on chromosome 16q22.1. This gene consists of 16 exons interwoven by 15 introns and is primarily localised within the cell membrane of epithelial cells [86]. While the extracellular domain of CDH1 is pivotal for cellular adhesion, its intracellular domain binds with the cytoskeleton via β-catenin, which triggers an array of intracellular signalling pathways [87]. A downregulation of E-cadherin has been observed in advanced malignancies, and E-cadherin loss has been shown to promote epithelial-to-mesenchymal transition (EMT), thus facilitating metastatic spread. The onset of EMT often marks the beginning of the malignant transformation in epithelial tumours, leading to decreased adherence of epithelial cells and facilitating their attachment to the basal membrane. A vital component of the EMT is the so-called “cadherin switch”, wherein reduced E-cadherin expression coincides with the acquisition of N-cadherin expression [86,87]. This transition promotes the mobility and invasiveness of cancer cells. EMT has also been associated with the upregulation of CDH2 and metalloproteinases and the downregulation of CDH1 within cancer cells. These molecular changes are reflected by extracellular matrix remodelling, enhanced migration and invasion, tumour stemness, and metastatic spread, thereby leading to the increased mortality of cancer patients. EMT is pivotal to cancer progression, drug resistance, and tumour stemness and provides a foundation for metastatic spread [88]. Therefore, the strategies to inhibit or prevent EMT are critical for impeding or ameliorating the progression of various human malignancies.

Immunohistochemical studies have demonstrated membranous E-cadherin expression in benign ovarian tumours and SBOTs. Notably, reduced E-cadherin expression has been shown to correlate with microinvasion in SBOTs [88,89]. The results of recent research involving cultured SBOT cells suggest that the downregulation of E-cadherin contributes to the progression of SBOTs towards invasive LGSCs. Interestingly, mucinous tumours appear to be more likely to express E-cadherin than serous tumours (62% vs 4%, *p* < 0.001) and have a lower likelihood of N-cadherin positivity (8% vs 68%, *p* < 0.001) [88–90]. The expression of E-cadherin protein was also found in inclusion cysts and benign, borderline, and malignant tumours of all stages, but it was reportedly absent in normal ovarian surface epithelium. It has thus been concluded that the loss or decrease of E-cadherin expression is a predictor of poorer overall survival in ovarian cancer and correlates with a higher tumour grade [90].

In conclusion, because of its involvement in cellular adhesion, EMT and intracellular signalling pathways, E-cadherin plays an essential role in cancer progression and spread and, as such, is a determinant of clinical outcomes in patients with various malignancies. Thus, pharmacological control of E-cadherin expression and related pathways appears to be a promising option for managing ovarian cancer.

### 4.15 BRCA 1/2

*BRCA1* and *BRCA2* mutations are specific genetic changes in tumour suppressor genes that appear in various instances of hereditary and some sporadic breast and ovarian cancers. If ovarian malignancy arises in a *BRCA1* or *BRCA2* carrier, it tends to be an HGSC that exhibits aggressive behaviour and presents at an advanced metastatic stage [91]. The occurrence of BOTs in women with *BRCA* mutations is uncommon. While BOTs have been identified in some *BRCA* mutation carriers, this occurrence can likely be attributed to the prevalence of these anomalies within the broader population [91].

Although mutations in the *BRCA1*, *BRCA2*, *RAD51C*, and *PALB2* genes were shown to be associated with an increased ovarian cancer risk, no such clear-cut relationship was found for BOTs. In fact, the prevalence of *BRCA* mutations in BOTs varies among different populations. In-depth research involving specific ethnicities or geographical groups might shed further light on the genetic background of BOTs. Nevertheless, several studies have demonstrated a link between *BRCA* mutations and BOTs. Specifically, two studies involving patients of Jewish heritage found that the occurrence of founder *BRCA1* and *BRCA2* mutations in women with BOTs was significantly lower than the corresponding rates in those with early-stage invasive ovarian carcinomas, with percentages of 2.2 and 4.3% versus 24.2 and 32%, respectively [91,92]. These findings are consistent with the results of studies conducted in Norway (including 190 patients with BOTs and 478 with ovarian cancer) and Canada (134 BOTs and 515 ovarian cancers), in which *BRCA1/2* mutations were detected in 4 and 11.7% of women with invasive cancers, respectively, but in none with BOTs [57,58]. Other smaller-scale studies have shown that *BRCA1/2* anomalies are found only sporadically in BOT patients, with the cumulative prevalence of *BRCA1* and *BRCA2* mutations of 1.3 and 0.2%, respectively [54]. In a Polish study, BOTs were diagnosed at a notably younger age than LGSCs (47.76 vs 54.25 years). When the results were stratified according to *BRCA1*, *BRCA2*, *RAD51C*, *PALB2*, and *CHEK2* mutational status, carriers of at least one of these anomalies turned out to be diagnosed with BOTs at a younger age than non-carriers (45 vs 49 years) [92,93].

In conclusion, a growing body of evidence suggests that the occurrence of *BRCA* mutations in patients with BOTs is generally lower than in those with invasive ovarian carcinomas. BOTs tend to be more closely associated with wild-type *BRCA* status, which implies that the pathogenesis of most of these tumours is not directly linked to the presence of *BRCA* mutations.

In summary, BOTs represent a group of ovarian tumours with a favourable prognosis. Preoperative diagnostics play a crucial role in guiding appropriate treatment decisions for patients. However, due to limited screening options and the reliance on markers like Ca125, HE4, and the ROMA algorithm for preoperative diagnosis, there is a need for new serum markers to aid in qualifying patients for surgical treatment. One promising candidate is TFF3, an oestrogen-regulated oncogene belonging to the trefoil factor family, or sFRP4, a modulator of the WNT signalling pathway. Various studies have suggested the potential utility of TFF3, particularly in diagnosing MBOT [94,95].

The primary imaging modality for preoperative diagnosis BOTs remains ultrasound, which helps identify tumour characteristics that may require urgent surgical intervention. In specific clinical situations, such as ovarian tumours concurrent with pregnancy, assessing adnexa becomes crucial, particularly in the first and early second trimesters. Therefore, upon suspicion of BOT during pregnancy based on the initial ultrasound, surgical removal of the ovarian mass should be considered to confirm histological diagnosis, unless contraindicated due to advanced gestational age [96]. Alternatively, an expectation management approach may be safe for managing BOT relapse during pregnancy or when suspicion arises in pregnant women at advanced gestational age [96].

Histopathological examination remains the cornerstone diagnostic tool for confirming BOTs. However, conventional histopathological parameters, including previously identified risk factors such as micropapillary patterns and microinvasion, have shown limited reliability in predicting recurrence risk. Given that BOTs primarily affect women of reproductive age, preserving fertility is paramount during treatment planning. Nevertheless, fertility-conserving surgeries and incomplete surgical staging have been associated with an increased risk of recurrence [97]. For patients planning pregnancies with early-stage BOTs eligible for conservative management, minimally invasive surgical techniques such as laparoscopy or robotic surgery are preferred. Additionally, the utilisation of mini-laparoscopic tools, such as needleoscopy, represents an intriguing and modern diagnostic option for patients desiring fertility preservation [97]. Although the standard surgical approach for BOTs resembles that of malignant ovarian tumours, lymphadenectomy during surgical staging is generally unnecessary. Conservative surgical treatment is suitable for young women with early-stage tumours (FIGO stage I–II), aiming to preserve fertility with close follow-up. However, adjuvant chemotherapy and/or radiotherapy have not shown convincing evidence of efficacy in improving prognosis to date.

Overall, BOTs demonstrate a favourable prognosis with low recurrence rates, even with conservative treatment. Essential aspects of ideal management include appropriate surgical staging, intraoperative tissue sampling, and diligent follow-up. Nevertheless, accurate predictive markers for relapse risk need to be identified. The balance between fertility-sparing surgery and the risk of recurrence remains a central concern in BOT management. Although fertility preservation is prioritized in reproductive-aged women, more aggressive surgery may be considered for postmenopausal patients [98].

## 5 Conclusions

In conclusion, BOTs often exhibit prolonged clinical dormancy before molecular triggers stimulate cell replication, potentially leading to carcinoma development or recurrence. Molecular analyses have revealed significant molecular and genetic similarities between serous BOTs and LGSCs, which points to a molecular shift in BOTs towards low-grade carcinoma via the ‘low-grade pathway’ characterised by mutations in the RAS/RAF/MEK/MAPK pathways. Additionally, specific molecular features associated with HGSCs can allow for the identification of a subgroup of BOTs prone to aggressive behaviour. The emergence of targeted therapies underscores the importance of detecting even minimal numbers of malignant cells within advanced-stage BOTs with additional driver mutations or clonal expansion. However, it is noteworthy that different mutations within the same pathway, such as KRAS, NRAS, and BRAF, may not uniformly respond to specific targeted therapies. Therefore, patients with diverse genetic mutations may require subset analysis in studies evaluating the efficacy of targeted interventions. While molecular studies significantly enhance our understanding of BOT pathogenesis, further research is essential to elucidate the relationship between ovarian neoplasms and extraneous diseases, identify precise prognostic indicators, and develop tailored therapeutic approaches.

## References

[1] Taylor H. Malignant and semi-malignant tumors of the ovary. Surg Gynecol Obstet. 1929;48:204–30.

[2] Hauptmann S, Friedrich K, Redline R, Avril S. Ovarian borderline tumors in the 2014 WHO classification: evolving concepts and diagnostic criteria. Virchows Arch. 2017;470(2):125–42. 10.1007/s00428-016-2040-8, Epub 2016 Dec 27 PMID: 28025670; PMCID: PMC5298321. PMC5298321 28025670

[3] Trillsch F, Ruetzel JD, Herwig U, Doerste U, Woelber L, Grimm D, et al. Surgical management and perioperative morbidity of patients with primary borderline ovarian tumor (BOT). J Ovarian Res. 2013 Jul;6(1):48. 10.1186/1757-2215-6-48, PMID: 23837881; PMCID: PMC3708757. PMC3708757 23837881

[4] Heintz AP, Odicino F, Maisonneuve P, Quinn MA, Benedet JL, Creasman WT, et al. Carcinoma of the ovary. FIGO 26th annual report on the results of treatment in gynecological cancer. Int J Gynaecol Obstet. 2006 Nov;95(Suppl 1):S161–92. 10.1016/S0020-7292(06)60033-7. PMID: 17161157. 17161157

[5] Wong HF, Low JJ, Chua Y, Busmanis I, Tay EH, Ho TH. Ovarian tumors of borderline malignancy: a review of 247 patients from 1991 to 2004. Int J Gynecol Cancer. 2007 Mar–Apr;17(2):342–9. 10.1111/j.1525-1438.2007.00864.x. Epub 2007 Mar 2 PMID: 17343573. 17343573

[6] Fischerova D, Zikan M, Dundr P, Cibula D. Diagnosis, treatment, and follow-up of borderline ovarian tumors. Oncologist. 2012;17(12):1515–33. 10.1634/theoncologist.2012-0139, Epub 2012 Sep 28 PMID: 23024155; PMCID: PMC3528384. PMC3528384 23024155

[7] Prat J. Pathology of borderline and invasive cancers. Best Pract Res Clin Obstet Gynaecol. 2017;41:15–30. 10.1016/j.bpobgyn.2016.08.007. 28277307

[8] Della Corte L, Mercorio A, Serafino P, Viciglione F, Palumbo M, De Angelis MC, et al. The challenging management of borderline ovarian tumors (BOTs) in women of childbearing age. Front Surg. 2022 Aug;9:973034. 10.3389/fsurg.2022.973034PMID: 36081590; PMCID: PMC9445208. PMC9445208 36081590

[9] Fang C, Zhao L, Chen X, Yu A, Xia L, Zhang P. The impact of clinicopathologic and surgical factors on relapse and pregnancy in young patients (≤40 years old) with borderline ovarian tumors. BMC Cancer. 2018 Nov;18(1):1147. 10.1186/s12885-018-4932-2PMID: 30463533; PMCID: PMC6249857. PMC6249857 30463533

[10] Raimondo D, Raffone A, Zakhari A, Maletta M, Vizzielli G, Restaino S, et al. The impact of hysterectomy on oncological outcomes in patients with borderline ovarian tumors: A systematic review and meta-analysis. Gynecol Oncol. 2022 Apr;165(1):184–91. 10.1016/j.ygyno.2022.01.019. Epub 2022 Jan 26 PMID: 35090745. 35090745

[11] Colombo N, Sessa C, du Bois A, Ledermann J, McCluggage WG, McNeish I, et al. ESMO-ESGO Ovarian Cancer Consensus Conference Working Group. ESMO-ESGO consensus conference recommendations on ovarian cancer: pathology and molecular biology, early and advanced stages, borderline tumours and recurrent disease†. Ann Oncol. 2019 May;30(5):672–705. 10.1093/annonc/mdz062. PMID: 31046081. 31046081

[12] Ronsini C, Restaino S, Budani MC, Porcelli G, Tiboni GM, Fanfani F. Fertility sparing treatment for bilateral borderline ovarian tumor: a case report and management strategy explication. Minerva Obstet Gynecol. 2023 Dec;75(6):583–7. 10.23736/S2724-606X.22.05115-6. Epub 2022 Oct 4 PMID: 36193828. 36193828

[13] Shih KK, Zhou Q, Huh J, Morgan JC, Iasonos A, Aghajanian C, et al. Risk factors for recurrence of ovarian borderline tumors. Gynecol Oncol. 2011 Mar;120(3):480–4. 10.1016/j.ygyno.2010.11.016, Epub 2010 Dec 10 PMID: 21146201; PMCID: PMC4843123. PMC4843123 21146201

[14] du Bois A, Ewald-Riegler N, de Gregorio N, Reuss A, Mahner S, Fotopoulou C, et al. Arbeitsgmeinschaft Gynäkologische Onkologie (AGO) Study Group. Borderline tumours of the ovary: A cohort study of the Arbeitsgmeinschaft Gynäkologische Onkologie (AGO) Study Group. Eur J Cancer. 2013 May;49(8):1905–14. 10.1016/j.ejca.2013.01.035. Epub 2013 Mar 13 PMID: 23490647. 23490647

[15] Śmiech M, Leszczyński P, Kono H, Wardell C, Taniguchi H. Emerging BRAF mutations in cancer progression and their possible effects on transcriptional networks. Genes (Basel). 2020 Nov;11(11):1342. 10.3390/genes11111342, PMID: 33198372; PMCID: PMC7697059. PMC7697059 33198372

[16] Peyssonnaux C, Eychène A. The Raf/MEK/ERK pathway: new concepts of activation. Biol Cell. 2001 Sep;93(1–2):53–62. 10.1016/s0248-4900(01)01125-x. PMID: 11730323. 11730323

[17] Chui MH, Kjaer SK, Frederiksen K, Hannibal CG, Wang TL, Vang R, et al. BRAFV600E -mutated ovarian serous borderline tumors are at relatively low risk for progression to serous carcinoma. Oncotarget. 2019 Dec;10(64):6870–8. 10.18632/oncotarget.27326.Erratum in: Oncotarget. 2021 Jun;12(13):1323-1324. PMID: 31839880; PMCID: PMC6901340. PMC6901340 31839880

[18] Shih IeM, Kurman RJ. Ovarian tumorigenesis: a proposed model based on morphological and molecular genetic analysis. Am J Pathol. 2004 May;164(5):1511–8. 10.1016/s0002-9440(10)63708-x. PMID: 15111296; PMCID: PMC1615664. PMC1615664 15111296

[19] Turashvili G, Grisham RN, Chiang S, DeLair DF, Park KJ, Soslow RA, et al. BRAFV600E mutations and immunohistochemical expression of VE1 protein in low-grade serous neoplasms of the ovary. Histopathology . 2018 Sep;73(3):438–43. 10.1111/his.13651, Epub 2018 Jun 22 PMID: 29770477; PMCID: PMC6105553. PMC6105553 29770477

[20] Zeppernick F, Ardighieri L, Hannibal CG, Vang R, Junge J, Kjaer SK, et al. BRAF mutation is associated with a specific cell type with features suggestive of senescence in ovarian serous borderline (atypical proliferative) tumors. Am J Surg Pathol. 2014 Dec;38(12):1603–11. 10.1097/PAS.0000000000000313. PMC4314953 25188864

[21] Wong KK, Tsang YT, Deavers MT, Mok SC, Zu Z, Sun C, et al. BRAF mutation is rare in advanced-stage low-grade ovarian serous carcinomas. Am J Pathol. 2010 Oct;177(4):1611–7. 10.2353/ajpath.2010.100212. Epub 2010 Aug 27. PMID: 20802181; PMCID: PMC2947258. PMC2947258 20802181

[22] Chen Y, Sun H, Deng Y, Ma Y, Huang H, Liu Y, et al. The clinical and genomic distinctions of Class1/2/3 BRAF-mutant colorectal cancer and differential prognoses. Biomark Res. 2023 Jan;11(1):11. 10.1186/s40364-022-00443-8, PMID: 36694231; PMCID: PMC9875443. PMC9875443 36694231

[23] Owsley J, Stein MK, Porter J, In GK, Salem M, O’Day S, et al. Prevalence of class I-III BRAF mutations among 114,662 cancer patients in a large genomic database. Exp Biol Med (Maywood). 2021 Jan;246(1):31–9. 10.1177/1535370220959657, Epub 2020 Oct 5 PMID: 33019809; PMCID: PMC7797994. PMC7797994 33019809

[24] Tsang YT, Deavers MT, Sun CC, Kwan SY, Kuo E, Malpica A, et al. KRAS (but not BRAF) mutations in ovarian serous borderline tumour are associated with recurrent low-grade serous carcinoma. J Pathol. 2013 Dec;231(4):449–56. 10.1002/path.4252, PMID: 24549645; PMCID: PMC4095747. PMC4095747 24549645

[25] Ohnishi K, Nakayama K, Ishikawa M, Ishibashi T, Yamashita H, Nakamura K, et al. Mucinous borderline ovarian tumors with BRAF(V600E) mutation may have low risk for progression to invasive carcinomas. Arch Gynecol Obstet. 2020 Aug;302(2):487–95. 10.1007/s00404-020-05638-8, Epub 2020 Jun 16 PubMed PMID: 32556513; PubMed Central PMCID: PMC7321901. PMC7321901 32556513

[26] Degirmenci U, Wang M, Hu J. Targeting aberrant RAS/RAF/MEK/ERK Signaling for cancer therapy. Cells. 2020 Jan;9(1):198. 10.3390/cells9010198, PMID: 31941155; PMCID: PMC7017232. PMC7017232 31941155

[27] Sadlecki P, Antosik P, Grzanka D, Grabiec M, Walentowicz-Sadlecka M. KRAS mutation testing in borderline ovarian tumors and low-grade ovarian carcinomas with a rapid, fully integrated molecular diagnostic system. Tumour Biol. 2017 Oct;39(10):1010428317733984. 10.1177/1010428317733984. PMID: 28992761. 28992761

[28] Ferreira A, Pereira F, Reis C, Oliveira MJ, Sousa MJ, Preto A. Crucial role of oncogenic KRAS mutations in apoptosis and autophagy regulation: therapeutic implications. Cells. 2022 Jul;11(14):2183. 10.3390/cells11142183, PMID: 35883626; PMCID: PMC9319879. PMC9319879 35883626

[29] Malpica A, Wong KK. The molecular pathology of ovarian serous borderline tumors. Ann Oncol . 2016 Apr;27(Suppl 1):i16–9. 10.1093/annonc/mdw089, PMID: 27141064; PMCID: PMC4852276. PMC4852276 27141064

[30] Zhu C, Guan X, Zhang X, Luan X, Song Z, Cheng X, et al. Targeting KRAS mutant cancers: from druggable therapy to drug resistance. Mol Cancer. 2022 Aug;21(1):159. 10.1186/s12943-022-01629-2, PMID: 35922812; PMCID: PMC9351107. PMC9351107 35922812

[31] Pązik M, Michalska K, Żebrowska-Nawrocka M, Zawadzka I, Łochowski M, Balcerczak E. Clinical significance of HRAS and KRAS genes expression in patients with non-small-cell lung cancer – preliminary findings. BMC Cancer. 2021 Feb;21(1):130. 10.1186/s12885-021-07858-w, PMID: 33549031; PMCID: PMC7866659. PMC7866659 33549031

[32] Kim HJ, Lee HN, Jeong MS, Jang SB. Oncogenic KRAS: signaling and drug resistance. Cancers (Basel). 2021 Nov;13(22):5599. 10.3390/cancers13225599, PMID: 34830757; PMCID: PMC8616169. PMC8616169 34830757

[33] Weber SM, Carroll SL. The role of r-ras proteins in normal and pathologic migration and morphologic change. Am J Pathol. 2021 Sep;191(9):1499–510. 10.1016/j.ajpath.2021.05.008, Epub 2021 Jun 7 PMID: 34111428; PMCID: PMC8420862. PMC8420862 34111428

[34] De Leo A, Santini D, Ceccarelli C, Santandrea G, Palicelli A, Acquaviva G et al. What is new on ovarian carcinoma: integrated morphologic and molecular analysis following the new 2020 World Health Organization Classification of female genital tumors. Diagnostics (Basel). 2021 Apr;11(4):697. 10.3390/diagnostics11040697, PMID: 33919741; PMCID: PMC8070731. PMC8070731 33919741

[35] Sheffels E, Kortum RL. The role of wild-type RAS in oncogenic RAS transformation. Genes (Basel). 2021 Apr;12(5):662. 10.3390/genes12050662, PMID: 33924994; PMCID: PMC8146411. PMC8146411 33924994

[36] Therachiyil L, Anand A, Azmi A, Bhat A, Korashy HM, Uddin S. Role of RAS signaling in ovarian cancer. F1000Res. 2022 Nov;11:1253. 10.12688/f1000research.126337.1, PMID: 36451660; PMCID: PMC9669513. PMC9669513 36451660

[37] Sadlecki P, Grzanka D, Grabiec M. Testing for NRAS mutations in serous borderline ovarian tumors and low-grade serous ovarian carcinomas. Dis Markers. 2018;2018:1497879. 10.1155/2018/1497879. PMC5845515 29682098

[38] Wiegand KC, Shah SP, Al-Agha OM, Zhao Y, Tse K, Zeng T, et al. ARID1A mutations in endometriosis-associated ovarian carcinomas. N Engl J Med. 2010 Oct;363(16):1532–43. 10.1056/NEJMoa1008433, Epub 2010 Sep 8 PMID: 20942669; PMCID: PMC2976679. PMC2976679 20942669

[39] Takeda T, Banno K, Okawa R, Yanokura M, Iijima M, Irie-Kunitomi H, et al. ARID1A gene mutation in ovarian and endometrial cancers (Review). Oncol Rep. 2016 Feb;35(2):607–13. 10.3892/or.2015.4421, Epub 2015 Nov 16 PMID: 26572704; PMCID: PMC4689482. PMC4689482 26572704

[40] Laban M, Chen X, Guo B. Seromucinous and mucinous borderline ovarian tumors: we need to know more. Reprod Sci. 2023 May;30(5):1684–5. 10.1007/s43032-022-01143-2. Epub 2022 Dec 6 PMID: 36474132. 36474132

[41] Prat J, D’Angelo E, Espinosa I. Ovarian carcinomas: at least five different diseases with distinct histological features and molecular genetics. Hum Pathol. 2018 Oct;80:11–27. 10.1016/j.humpath.2018.06.018. Epub 2018 Jun 23 PMID: 29944973. 29944973

[42] Kurman RJ, Shih IeM. Seromucinous tumors of the ovary. what’s in a name? Int J Gynecol Pathol. 2016 Jan;35(1):78–81. 10.1097/PGP.0000000000000266, PMID: 26598986; PMCID: PMC5512580. PMC5512580 26598986

[43] Ayhan A, Mao TL, Seckin T, Wu CH, Guan B, Ogawa H, et al. Loss of ARID1A expression is an early molecular event in tumor progression from ovarian endometriotic cyst to clear cell and endometrioid carcinoma. Int J Gynecol Cancer. 2012 Oct;22(8):1310–5. 10.1097/IGC.0b013e31826b5dcc, PMID: 22976498; PMCID: PMC3460070. PMC3460070 22976498

[44] Nagamine M, Mikami Y. Ovarian seromucinous tumors: pathogenesis, morphologic spectrum, and clinical issues. Diagnostics (Basel). 2020 Jan;10(2):77. 10.3390/diagnostics10020077, PMID: 32023964; PMCID: PMC7168900. PMC7168900 32023964

[45] Maeda D, Shih IeM. Pathogenesis and the role of ARID1A mutation in endometriosis-related ovarian neoplasms. Adv Anat Pathol. 2013 Jan;20(1):45–52. 10.1097/PAP.0b013e31827bc24d, Review. PubMed PMID: 23232571; PubMed Central PMCID: PMC3523307. PMC3523307 23232571

[46] Si X, Xu F, Xu F, Wei M, Ge Y, Chenge S. CADM1 inhibits ovarian cancer cell proliferation and migration by potentially regulating the PI3K/Akt/mTOR pathway. Biomed Pharmacother. 2020 Mar;123:109717. 10.1016/j.biopha.2019.109717. Epub 2019 Dec 25 PMID: 31865146. 31865146

[47] Vallath S, Sage EK, Kolluri KK, Lourenco SN, Teixeira VS, Chimalapati S, et al. CADM1 inhibits squamous cell carcinoma progression by reducing STAT3 activity. Sci Rep. 2016 Apr;6:24006. 10.1038/srep24006, PMID: 27035095; PMCID: PMC4817512. PMC4817512 27035095

[48] Li H, Gao J, Zhang S. Functional and clinical characteristics of cell adhesion molecule CADM1 in cancer. Front Cell Dev Biol. 2021 Jul;9:714298. 10.3389/fcell.2021.714298, PMID: 34395444; PMCID: PMC8361327. PMC8361327 34395444

[49] Wu D, Lei Y, Liu Q, Hu H, Li H, Xie L, et al. Characterization and clinical significance of the CADM1/HER2/STAT3 axis in serous ovarian tumors. Medicine (Baltimore). 2021 Feb;100(8):e23777. 10.1097/MD.0000000000023777, PMID: 33663040; PMCID: PMC7909124. PMC7909124 33663040

[50] Samuels Y, Ericson K. Oncogenic PI3K and its role in cancer. Curr Opin Oncol. 2006 Jan;18(1):77–82. 10.1097/01.cco.0000198021.99347.b9. PMID: 16357568. 16357568

[51] Levine DA, Bogomolniy F, Yee CJ, Lash A, Barakat RR, Borgen PI, et al. Frequent mutation of the PIK3CA gene in ovarian and breast cancers. Clin Cancer Res. 2005 Apr;11(8):2875–8. 10.1158/1078-0432.CCR-04-2142. PMID: 15837735. 15837735

[52] Ishibashi T, Nakayama K, Razia S, Ishikawa M, Nakamura K, Yamashita H, et al. High frequency of PIK3CA mutations in low-grade serous ovarian carcinomas of japanese patients. Diagnostics (Basel). 2019 Dec;10(1):13. 10.3390/diagnostics10010013, PMID: 31892193; PMCID: PMC7168240. PMC7168240 31892193

[53] Nonomura Y, Nakayama K, Nakamura K, Razia S, Yamashita H, Ishibashi T, et al. Ovarian endometrioid and clear cell carcinomas with low prevalence of microsatellite instability: a unique subset of ovarian carcinomas could benefit from combination therapy with immune checkpoint inhibitors and other anticancer agents. Healthcare (Basel). 2022 Apr;10(4):694. 10.3390/healthcare10040694, PMID: 35455871; PMCID: PMC9032309. PMC9032309 35455871

[54] Stolarova L, Kleiblova P, Janatova M, Soukupova J, Zemankova P, Macurek L, et al. CHEK2 germline variants in cancer predisposition: stalemate rather than checkmate. Cells. 2020 Dec;9(12):2675. 10.3390/cells9122675, PMID: 33322746; PMCID: PMC7763663. PMC7763663 33322746

[55] Kleiblova P, Stolarova L, Krizova K, Lhota F, Hojny J, Zemankova P, et al. Identification of deleterious germline CHEK2 mutations and their association with breast and ovarian cancer. Int J Cancer. 2019 Oct;145(7):1782–97. 10.1002/ijc.32385. Epub 2019 May 20 PMID: 31050813. 31050813

[56] Ogrodniczak A, Menkiszak J, Gronwald J, Tomiczek-Szwiec J, Szwiec M, Cybulski C, et al. Association of recurrent mutations in BRCA1, BRCA2, RAD51C, PALB2, and CHEK2 with the risk of borderline ovarian tumor. Hered Cancer Clin Pract. 2022 Mar;20(1):11. 10.1186/s13053-022-00218-0, PMID: 35313928; PMCID: PMC8935754. PMC8935754 35313928

[57] Öfverholm A, Törngren T, Rosén A, Arver B, Einbeigi Z, Haraldsson K, et al. Extended genetic analysis and tumor characteristics in over 4600 women with suspected hereditary breast and ovarian cancer. BMC Cancer. 2023 Aug;23(1):738. 10.1186/s12885-023-11229-y, PMID: 37563628; PMCID: PMC10413543. PMC10413543 37563628

[58] Li J. Dysregulated expression of claudins in cancer. Oncol Lett. 2021 Sep;22(3):641. 10.3892/ol.2021.12902. Epub 2021 Jul 7. PMID: 34386063; PMCID: PMC8298996. PMC8298996 34386063

[59] Wang DW, Zhang WH, Danil G, Yang K, Hu JK. The role and mechanism of claudins in cancer. Front Oncol. 2022 Dec;12:1051497. 10.3389/fonc.2022.1051497, PMID: 36620607; PMCID: PMC9818346. PMC9818346 36620607

[60] Curry EW, Stronach EA, Rama NR, Wang YY, Gabra H, El-Bahrawy MA. Molecular subtypes of serous borderline ovarian tumor show distinct expression patterns of benign tumor and malignant tumor-associated signatures. Mod Pathol. 2014 Mar;27(3):433–42. 10.1038/modpathol.2013.13. Epub 2013 Aug 16. PMID: 23948749. 23948749

[61] Zhu Y, Brännström M, Janson PO, Sundfeldt K. Differences in expression patterns of the tight junction proteins,claudin 1, 3, 4 and 5, in human ovarian surface epithelium as compared to epithelia in inclusion cysts and epithelial ovarian tumours. Int J Cancer. 2006 Apr;118(8):1884–91. 10.1002/ijc.21506. PMID: 16287068. 16287068

[62] Maihle NJ, Baron AT, Barrette BA, Boardman CH, Christensen TA, Cora EM, et al. EGF/ErbB receptor family in ovarian cancer. Cancer Treat Res. 2002;107:247–58. 10.1007/978-1-4757-3587-1_11. PMID: 11775453. 11775453

[63] Arteaga CL, Engelman JA. ERBB receptors: from oncogene discovery to basic science to mechanism-based cancer therapeutics. Cancer Cell. 2014 Mar;25(3):282–303. 10.1016/j.ccr.2014.02.025, PMID: 24651011; PMCID: PMC4018830. PMC4018830 24651011

[64] Farley J, Fuchiuji S, Darcy KM, Tian C, Hoskins WJ, McGuire WP, et al. Associations between ERBB2 amplification and progression-free survival and overall survival in advanced stage, suboptimally-resected epithelial ovarian cancers: a gynecologic oncology group study. Gynecol Oncol. 2009 Jun;113(3):341–7. 10.1016/j.ygyno.2009.02.009, Epub 2009 Mar 9 PMID: 19272639; PMCID: PMC6944288. PMC6944288 19272639

[65] Mackenzie R, Kommoss S, Winterhoff BJ, Kipp BR, Garcia JJ, Voss J, et al. Targeted deep sequencing of mucinous ovarian tumors reveals multiple overlapping RAS-pathway activating mutations in borderline and cancerous neoplasms. BMC Cancer. 2015 May;15:415. 10.1186/s12885-015-1421-8, PMID: 25986173; PMCID: PMC4494777. PMC4494777 25986173

[66] Sasamori H, Nakayama K, Razia S, Yamashita H, Ishibashi T, Ishikawa M, et al. Mutation profiles of ovarian seromucinous borderline tumors in japanese patients. Curr Oncol. 2022 May;29(5):3658–67. 10.3390/curroncol29050294, PMID: 35621684; PMCID: PMC9139622. PMC9139622 35621684

[67] Murakami K, Kotani Y, Nakai H, Matsumura N. Endometriosis-associated ovarian cancer: the origin and targeted therapy. Cancers (Basel). 2020 Jun;12(6):1676. 10.3390/cancers12061676, PMID: 32599890; PMCID: PMC7352633. PMC7352633 32599890

[68] Ryland GL, Doyle MA, Goode D, Boyle SE, Choong DY, Rowley SM, et al. Loss of heterozygosity: what is it good for? BMC Med Genomics. 2015 Aug;8:45. 10.1186/s12920-015-0123-z, PMID: 26231170; PMCID: PMC4522148. PMC4522148 26231170

[69] Testa U, Pelosi E, Castelli G. Colorectal cancer: genetic abnormalities, tumor progression, tumor heterogeneity, clonal evolution and tumor-initiating cells. Med Sci (Basel). 2018 Apr;6(2):31. 10.3390/medsci6020031, PMID: 29652830; PMCID: PMC6024750. PMC6024750 29652830

[70] Russo A, Czarnecki AA, Dean M, Modi DA, Lantvit DD, Hardy L, et al. PTEN loss in the fallopian tube induces hyperplasia and ovarian tumor formation. Oncogene. 2018 Apr;37(15):1976–90. 10.1038/s41388-017-0097-8, Epub 2018 Jan 25 PMID: 29367766; PMCID: PMC6472269. PMC6472269 29367766

[71] Fusco N, Sajjadi E, Venetis K, Gaudioso G, Lopez G, Corti C, et al. PTEN alterations and their role in cancer management: are we making headway on precision medicine? Genes (Basel). 2020 Jun;11(7):719. 10.3390/genes11070719, PMID: 32605290; PMCID: PMC7397204. PMC7397204 32605290

[72] Csolle MP, Ooms LM, Papa A, Mitchell CA. PTEN and Other PtdIns(3,4,5)P3 lipid phosphatases in breast cancer. Int J Mol Sci. 2020 Dec;21(23):9189. 10.3390/ijms21239189, PMID: 33276499; PMCID: PMC7730566. PMC7730566 33276499

[73] Driva TS, Schatz C, Haybaeck J. Endometriosis-Associated ovarian carcinomas: How PI3K/AKT/mTOR pathway affects their pathogenesis. Biomolecules. 2023 Aug;13(8):1253. 10.3390/biom13081253, PMID: 37627318; PMCID: PMC10452661. PMC10452661 37627318

[74] Wei JJ, William J, Bulun S. Endometriosis and ovarian cancer: a review of clinical, pathologic, and molecular aspects. Int J Gynecol Pathol. 2011 Nov;30(6):553–68. 10.1097/PGP.0b013e31821f4b85, PMID: 21979592; PMCID: PMC4130217. PMC4130217 21979592

[75] Liao X, Zhu W, Zhou J, Li H, Xu X, Zhang B, et al. Repetitive DNA sequence detection and its role in the human genome. Commun Biol. 2023 Sep;6(1):954. 10.1038/s42003-023-05322-y, PMID: 37726397; PMCID: PMC10509279. PMC10509279 37726397

[76] Boland CR, Goel A. Microsatellite instability in colorectal cancer. Gastroenterology. 2010;138(6):2073–87.e3. 10.1053/j.gastro.2009.12.064, PMID: 20420947; PMCID: PMC3037515. PMC3037515 20420947

[77] Zhao S, Chen L, Zang Y, Liu W, Liu S, Teng F, et al. Endometrial cancer in Lynch syndrome. Int J Cancer. 2022 Jan;150(1):7–17. 10.1002/ijc.33763. Epub 2021 Sep 9 PMID: 34398969. 34398969

[78] Sanz Casla MT, Vidaurreta Lazaro M, Almansa de Lara I, Tresserra F, Lopez Marin L, Maestro ML, et al. Role of microsatellite instability in borderline ovarian tumors. Anticancer Res. 2003 Nov–Dec;23(6D):5139–41. PMID: 14981979. 14981979

[79] Vergara D, Tinelli A, Martignago R, Malvasi A, Chiuri VE, Leo G. Biomolecular pathogenesis of borderline ovarian tumors: focusing target discovery through proteogenomics. Curr Cancer Drug Targets. 2010 Feb;10(1):107–16. 10.2174/156800910790980269. PMID: 20088785. 20088785

[80] Milanesio MC, Giordano S, Valabrega G. Clinical implications of DNA repair defects in high-grade serous ovarian carcinomas. Cancers (Basel). 2020 May;12(5):1315. 10.3390/cancers12051315, PMID: 32455819; PMCID: PMC7281678. PMC7281678 32455819

[81] Shih IeM, Kurman RJ. Molecular pathogenesis of ovarian borderline tumors: new insights and old challenges. Clin Cancer Res. 2005 Oct;11(20):7273–9. 10.1158/1078-0432.CCR-05-0755. PMID: 16243797. 16243797

[82] Mbom BC, Nelson WJ, Barth A. β-catenin at the centrosome: discrete pools of β-catenin communicate during mitosis and may co-ordinate centrosome functions and cell cycle progression. Bioessays. 2013 Sep;35(9):804–9. 10.1002/bies.201300045, Epub 2013 Jun 27 PMID: 23804296; PMCID: PMC3983869. PMC3983869 23804296

[83] Nguyen VHL, Hough R, Bernaudo S, Peng C. Wnt/β-catenin signalling in ovarian cancer: Insights into its hyperactivation and function in tumorigenesis. J Ovarian Res. 2019 Dec;12(1):122. 10.1186/s13048-019-0596-z, PMID: 31829231; PMCID: PMC6905042. PMC6905042 31829231

[84] Varga J, Reviczká A, Háková H, Švajdler P, Rabajdová M, Ostró A. Predictive factors of endometriosis progression into ovarian cancer. J Ovarian Res. 2022 Jan;15(1):5. 10.1186/s13048-021-00940-8, PMID: 35012617; PMCID: PMC8751310. PMC8751310 35012617

[85] Loh CY, Chai JY, Tang TF, Wong WF, Sethi G, Shanmugam MK, et al. The E-Cadherin and n-cadherin switch in epithelial-to-mesenchymal transition: signaling, therapeutic implications, and challenges. Cells. 2019 Sep;8(10):1118. 10.3390/cells8101118, PMID: 31547193; PMCID: PMC6830116. PMC6830116 31547193

[86] Pećina-Slaus N. Tumor suppressor gene E-cadherin and its role in normal and malignant cells. Cancer Cell Int. 2003 Oct;3(1):17. 10.1186/1475-2867-3-17, PMID: 14613514; PMCID: PMC270068. PMC270068 14613514

[87] Altamura C, Greco MR, Carratù MR, Cardone RA, Desaphy JF. Emerging roles for ion channels in ovarian cancer: pathomechanisms and pharmacological treatment. Cancers (Basel). 2021 Feb;13(4):668. 10.3390/cancers13040668, PMID: 33562306; PMCID: PMC7914442. PMC7914442 33562306

[88] Woo MM, Salamanca CM, Miller M, Symowicz J, Leung PC, Oliveira C, et al. Serous borderline ovarian tumors in long-term culture: phenotypic and genotypic distinction from invasive ovarian carcinomas. Int J Gynecol Cancer. 2008 Nov–Dec;18(6):1234–47. 10.1111/j.1525-1438.2007.01171.x. Epub 2008 Jan 23 PMID: 18217967. 18217967

[89] Dai C, Cao J, Zeng Y, Xu S, Jia X, Xu P. E-cadherin expression as a prognostic factor in patients with ovarian cancer: a meta-analysis. Oncotarget. 2017 Jun;8(46):81052–61. 10.18632/oncotarget.18898, PMID: 29113366; PMCID: PMC5655261. PMC5655261 29113366

[90] Sekine M, Nishino K, Enomoto T. Differences in ovarian and other cancers risks by population and BRCA mutation location. Genes (Basel). 2021 Jul;12(7):1050. 10.3390/genes12071050, PMID: 34356066; PMCID: PMC8303997. PMC8303997 34356066

[91] Nayyar N, Lakhwani P, Goel A, Pande PK, Kumar K. Management of borderline ovarian tumors-still a gray zone. Indian J Surg Oncol. 2017 Dec;8(4):607–14. 10.1007/s13193-017-0697-3. Epub 2017 Aug 25. PMID: 29203995; PMCID: PMC5705520. PMC5705520 29203995

[92] Łukomska A, Menkiszak J, Gronwald J, Tomiczek-Szwiec J, Szwiec M, Jasiówka M, et al. Recurrent mutations in BRCA1, BRCA2, RAD51C, PALB2 and CHEK2 in polish patients with ovarian cancer. Cancers (Basel). 2021 Feb;13(4):849. 10.3390/cancers13040849, PMID: 33670479; PMCID: PMC7921976. PMC7921976 33670479

[93] Turan H, Vitale SG, Kahramanoglu I, Della Corte L, Giampaolino P, Azemi A, et al. Diagnostic and prognostic role of TFF3, Romo-1, NF-кB and SFRP4 as biomarkers for endometrial and ovarian cancers: a prospective observational translational study. Arch Gynecol Obstet. 2022 Dec;306(6):2105–14. 10.1007/s00404-022-06563-8, Epub 2022 Apr 24 PMID: 35461390; PMCID: PMC9633503. PMC9633503 35461390

[94] El-Balat A, Schmeil I, Karn T, Becker S, Sänger N, Holtrich U, et al. TFF3 expression as stratification marker in borderline epithelial tumors of the ovary. Pathol Oncol Res. 2018 Apr;24(2):277–82. 10.1007/s12253-017-0240-4. Epub 2017 May 3 PMID: 28470574. 28470574

[95] Werner Rönnerman E, Pettersson D, Nemes S, Dahm-Kähler P, Kovács A, Karlsson P, et al. Trefoil factor family proteins as potential diagnostic markers for mucinous invasive ovarian carcinoma. Front Oncol. 2023;12:1112152 10.3389/fonc.2022.1112152 PMC9932968 36818673

[96] Iavazzo C, Gkegkes ID. Expectant management of borderline ovarian tumor during pregnancy. Arch Gynecol Obstet. 2021 Dec;304(6):1623–4. 10.1007/s00404-021-06247-9. Epub 2021 Oct 7 PMID: 34622339. 34622339

[97] Gueli Alletti S, Rossitto C, Perrone E, Cianci S, De Blasis I, Fagotti A, et al. Needleoscopic conservative staging of borderline ovarian tumor. J Minim Invasive Gynecol. 2017 May–Jun;24(4):529–30. 10.1016/j.jmig.2016.10.009. Epub 2016 Oct 27 PMID: 27989810. 27989810

[98] Pecorino B, Laganà AS, Mereu L, Ferrara M, Carrara G, Etrusco A, et al. Evaluation of Borderline ovarian tumor recurrence rate after surgery with or without fertility-sparing approach: results of a retrospective analysis. Healthcare (Basel). 2023 Jul;11(13):1922. 10.3390/healthcare11131922, PMID: 37444757; PMCID: PMC10341047. PMC10341047 37444757

